# Impact of anticoagulation management following endovascular therapy on prognosis of patients with atrial fibrillation and acute ischemic stroke

**DOI:** 10.3389/fcvm.2025.1622665

**Published:** 2026-01-15

**Authors:** Yu Zhu, Xueqi Lin, Kadiyan Aierken, Yajie Zhu, Jiahui Zhou, Jing Gao, Hongling Zhao, Tao Wang, Shijun Li

**Affiliations:** 1 Department of Cardiology, Central Hospital of Dalian University of Technology (Dalian Municipal Central Hospital), Dalian, China; 2 China Medical University, Shenyang, China; 3 Dalian Medical University, Dalian, China; 4 Department of Neurology, Central Hospital of Dalian University of Technology (Dalian Municipal Central Hospital), Dalian, China

**Keywords:** acute ischemic stroke, atrial fibrillation, endovascular therapy, hemorrhagic transformation, oral anticoagulants

## Abstract

**Objective:**

This study evaluates the impact of early oral anticoagulant (OAC) initiation at hospital discharge on functional and safety outcomes in atrial fibrillation (AF)-related acute ischemic stroke (AIS) patients undergoing endovascular therapy (EVT).

**Methods:**

AF patients undergoing EVT of symptom onset in AIS were included in this study. Patients were grouped by postoperative anticoagulation status. The patients were regularly followed up 90 days and 1 year after discharge. The primary outcome measure for assessing prognosis is the modified Rankin Scale (mRS) score. The secondary evaluation indicators were the occurrence of recurrent ischemic stroke/systemic embolism (IS/SE) outcomes, safety outcomes, and all-cause mortality outcomes within 1 year. The differences in prognostic indicators between the two groups were compared by combining PSM.

**Results:**

Among the 296 eligible patients, 113 (38.18%) received anticoagulation at discharge, while 183 (61.82%) did not. Before PSM, the anticoagulation cohort exhibited markedly elevated rates of favorable functional outcomes at 90 days post-discharge (mRS 0–2: 60.18% vs. 25.14%, *P* < 0.001) and at 1 year (mRS 0–2: 64.60% vs. 30.05%, *P* < 0.001), along with a lower all-cause mortality rate within 1 year (16.81% vs. 44.26%, *P* < 0.001). After PSM, the results demonstrated that the anticoagulation group had elevated rates of favorable functional outcomes at 90 days (55.81% vs. 27.91%, *P* < 0.001) and at 1 year (60.47% vs. 30.23%, *P* < 0.001). The anticoagulation group had a lower all-cause mortality rate at both 90-day (11.63% vs. 40.70%, *P* < 0.001) and 1-year follow-up (17.44% vs. 50%, *P* < 0.001). Statistical analysis revealed no significant intergroup differences in terms of IS/SE recurrence rates, safety outcomes. Multivariate logistic regression modeling identified OAC therapy upon discharge as an independent predictor of improved 90-days (OR = 4.478, 95% CI: 1.122–17.874, *P* = 0.034) and 1 year (OR = 4.168, 95% CI: 1.118–5.542, *P* = 0.033) functional recovery among patients.

**Conclusion:**

In patients with AF complicated by stroke who underwent EVT and were at no high risk for severe bleeding, OAC therapy is associated with improved functional and mortality outcomes compared with those not receiving OAC. The benefit remained statistically significant following PSM to adjust for intergroup disparities.

## 1 Introduction

Atrial fibrillation (AF) is the most prevalent cardiac arrhythmia in adults, confers a 3–5 fold increased risk of acute ischemic stroke (AIS) compared to non-AF populations which is accompanied by significantly higher mortality rates and worse functional outcomes (1–3). Therefore, long-term oral anticoagulation (OAC) was recommended for secondary prevention (4–8). However, the optimal time of OAC initiation post-AIS remains contentious, particularly balancing thromboembolic recurrence risk against hemorrhagic risk (9–12).

Endovascular therapy (EVT) demonstrates substantial clinical benefit in AF-related AIS patients with large vessel occlusion, particularly within 24 h of symptom onset (13), and enhanced therapeutic efficacy observed in patients with salvage brain tissue during extended time windows of 6–24 h (14). EVT can restore blood flow to occluded arteries, but reperfusion is more likely to lead to hemorrhagic transformation (HT) (15). The suboptimal utilization of OAC both during hospitalization and post-discharge periods may be attributed to heightened post-EVT hemorrhagic transformation risk and stroke severity. Whether timely anticoagulation management after EVT can improve outcomes in all patients with AF and AIS still requires further corroboration from relevant studies. Therefore, this study will utilize propensity score matching (PSM) to analyze the safety and efficacy of early postoperative anticoagulation in these patients, specifically investigating its association with functional outcomes, recurrent ischemic stroke/systemic embolism (IS/SE), safety, and all-cause mortality.

## 2 Materials and methods

### 2.1 Study population

This study enrolled patients diagnosed with AF complicated by AIS who underwent EVT (including mechanical thrombectomy, arterial thrombolysis, stenting, and balloon dilation) within 24 h of stroke onset at Dalian Central Hospital between January 2020 and December 2023. Exclusion criteria: (1) Patients with valvular cardiac pathology or rheumatic heart disease; (2) Patients with a history of intracerebral hemorrhage and recent (3 months) active visceral hemorrhage; (3) Patients who were discharged against medical advice, transferred to other institutions, or died during hospitalization due to severe complications; (4) Patients with severe liver and kidney insufficiency and hematologic diseases; (5) Patients who discontinued follow-up; (6) Patients demonstrating non-adherence to anticoagulation therapy during follow-up. This research received approval from the Human Ethics Committee and the Medical Research Council of Dalian Central Hospital, and written informed consent was secured from all patients or their guardians.

### 2.2 Collection of information

General clinical data were collected including: age, gender, body mass index (BMI), smoking, drinking, underlying diseases (hypertension, diabetes mellitus, coronary heart disease, heart failure, hyperlipidemia), type of AF, history of previous transient ischemic attack (TIA) or stroke, use of OAC before stroke, CHA2DS2-VASc score, National institutes of health stroke scale (NIHSS) score (16), HAS-BLED score, modified Rankin Scale (mRS) score (17), N-terminal pro-B-type natriuretic peptide (NT-proBNP), left atrial diameter (LAD), left ventricular ejection fraction (LVEF), D-dimer level, and creatinine level. And collected surgery-related data: time from onset to femoral artery puncture, combined with intravenous thrombolysis (IVT), surgical methods (mechanical thrombectomy, arterial thrombolysis, stent placement and balloon dilation), modified thrombolysis in cerebral ischemia (mTICI) grading, combined with multiple vascular occlusions, combined with severe intracranial atherosclerotic stenosis (>70%), combined with tandem vascular lesions, infarct size, and postoperative hemorrhagic transformation (HT). Any form of hemorrhage detected within the infarct area through imaging examinations is defined as HT with performing a follow-up head CT within 24 h after EVT.

### 2.3 Treatment group

All patients received conventional treatments, including lipid-lowering and plaque-stabilizing therapies, adjunctive therapy for improving cerebral blood flow perfusion, blood pressure and blood glucose stabilization, and complication control after EVT, following hospital admission. The appropriate antithrombotic regimen was selected in the hospital after evaluating the risk of bleeding.

All patients were divided into the following two groups according to whether the patients received long-term OAC treatment at the time of discharge: (1) The anticoagulation group was defined as patients who continued to receive long-term OAC treatment after risk assessment by the attending physician at discharge, including OAC monotherapy or OAC combined with antiplatelet therapy; (2) The non-anticoagulation group was defined as patients who did not receive OAC therapy after assessment by the attending physician at discharge and did not initiate delayed anticoagulation during the follow-up period.

### 2.4 Follow-up and prognostic evaluation indicators

All patients completed the follow-up at 90 days and 1 year after discharge, and the follow-up data came from the stroke prevention and control cloud platform of Dalian Central hospital, and the mRS score at 90 days, mRS score at 1-year, anticoagulant medication were collected and recorded. The mRS score refers to the modified Rankin Scale score, with a total of 6 points. It provides a quantitative assessment of neurological functional recovery in stroke patients. A score of 0–2 indicates no significant symptoms to minor sequelae, 3–5 indicates moderate to severe disability due to sequelae, and a score of 6 indicates death (17). Additionally, record the occurrence of recurrent ischemic stroke/systemic embolism (IS/SE) outcomes, safety outcomes (including symptomatic intracerebral hemorrhage or major bleeding at critical organ sites), and all-cause mortality outcomes within 1 year.

The main prognostic indicators were evaluated according to the functional outcome of the patients at follow-up, with an mRS score of ≤2 defined as favorable functional outcomes. Other indicators for assessing prognosis include the occurrence of other adverse outcomes within 1 year.

### 2.5 Statistical analysis

For normally distributed continuous variables, independent samples *t*-tests were employed, with results presented as (Mean ± SD). Non-normally distributed data were analyzed using Mann–Whitney *U* tests, reported as median (IQR). Categorical variables were compared between groups using *χ*^2^ tests and reported as percentages. PSM was used to control for potential confounders, and propensity scores were estimated using logistic regression with a matching ratio of 1:1 and a caliper value of 0.02. The variables of PSM were factors that significantly differed from baseline data between groups or confounders that may affect outcomes, and differences were compared between groups after matching. Using covariates including admission NIHSS score, HAS-BLED score, LVEF, post-operative HT, infarct size, discharge NIHSS score, discharge mRS score, AF subtype, and surgical approach, a 1:1 matching was performed between the two groups.

Post-PSM cohort stratification was performed using median-based cutoffs: age (<72 vs. ≥72 years), baseline NIHSS score (<16 vs. ≥16), and atrial fibrillation classification [atrial fibrillation detected after stroke (AFDAS) vs. known atrial fibrillation (KAF)]. Subgroup analyses stratified by age, sex, AF classification, admission NIHSS score, and IVT coadministration status were conducted to evaluate the relationship of anticoagulant therapy with 1-year functional outcomes and recurrent IS/SE events. All baseline admission data were included in a univariate analysis. Subsequently, factors significantly associated with a good functional outcome (mRS 0–2) at a significance level of *P* < 0.05 were incorporated into a multivariate analysis to calculate the odds ratio (OR) and 95% confidence interval (CI) for the association between anticoagulant therapy and functional outcomes. The analysis and processing of the data in this study was completed in SPSS 27.0 software and Zstatsv1.0 software.

## 3 Results

### 3.1 Baseline characteristics of patients

This study included 296 patients with complete follow-up, stratified into anticoagulation (*n* = 113, 38.18%) and non-anticoagulation (*n* = 183, 61.82%) groups. Percentage of patients who were on anticoagulants at the time of hospital discharge was 5.31% (*n* = 6) on Edoxaban, 60.18% (*n* = 68) on Rivaroxaban and 34.51% (*n* = 39) were on Dabigatran Etexilate (Supplementary Table S2). Additional details regarding the timing of anticoagulation initiation (Supplementary Table S1) and antiplatelet medications (Supplementary Table S3) are provided in the Supplementary Materials. The median admission NIHSS scores (16 vs. 18, *P* = 0.043), discharge NIHSS scores (6 vs. 13, *P* < 0.001), discharge mRS scores (3 vs. 4, *P* < 0.001) and the incidence of postoperative HT (12.39% vs. 31.69%, *P* < 0.001) were significantly higher in the non-anticoagulation group. Additionally, the anticoagulation group had a higher LVEF (54.81% vs. 53.32%, *P* = 0.026) than the non-anticoagulation group. Furthermore, there was a significant difference in infarct size between the two groups (*P* = 0.002), with the anticoagulation group demonstrating smaller infarct areas (Table 1).

**Table 1:** Baseline characteristics of the anticoagulation vs. non-anticoagulation groups before PSM.

Characteristics	Anticoagulation group (*n* = 113)	Non-anticoagulation group (*n* = 183)	*P* value
Age, years, mean ± SD	72.48 ± 8.83	73.42 ± 8.67	0.370
Male, *n* (%)	68 (60.18)	105 (57.38)	0.635
BMI, kg/m^2^, mean ± SD	25.78 ± 3.71	25.00 ± 3.27	0.059
Medical history, *n* (%)			
Smoking	42 (37.17)	65 (35.52)	0.774
Drinking	28 (24.78)	44 (24.04)	0.886
Stroke/TIA	23 (20.35)	44 (24.04)	0.461
Hypertension	72 (63.72)	131 (71.58)	0.157
Diabetes mellitus	30 (26.55)	60 (32.79)	0.257
Coronary heart disease	23 (20.35)	27 (14.75)	0.212
Hyperlipemia	40 (35.4)	72 (39.34)	0.496
Heart failure	20 (17.70)	33 (18.03)	0.942
Types of AF, *n* (%)			
Paroxysmal AF (classification by duration)	22 (19.47)	28 (15.30)	0.352
Non-paroxysmal AF (classification by duration)	91 (80.53)	155 (84.70)	
KAF (classification by diagnosis time)	81 (71.68)	138 (75.41)	0.477
AFDAS (classification by diagnosis time)	32 (28.32)	45 (24.59)	
Clinically relevant baseline characteristics			
History of pre-stroke OAC use, *n* (%)	12 (10.62)	11 (6.01)	0.150
CHA2DS2-VASc score, median (IQR)	5.00 (4.00, 6.00)	5.00 (4.00, 6.00)	0.161
HAS-BLED score, median (IQR)	2.00 (2.00, 2.00)	2.00 (2.00, 3.00)	<0.001
Admission NIHSS score, median (IQR)	16.00 (10.50, 20.00)	18.00 (13.00, 23.00)	0.043
Admission mRS score, median (IQR)	4.00 (4.00, 5.00)	5.00 (4.00, 5.00)	0.183
Discharge NIHSS score, median (IQR)	6.00 (2.00, 12.00)	13.00 (6.00, 19.00)	<0.001
Discharge mRS score, median (IQR)	3.00 (1.00, 4.00)	4.00 (3.00, 5.00)	<0.001
NT-proBNP, pg/mL, median (IQR)	1,411.28 (820.00, 2,468.90)	1,739.90 (826.00, 2,891.20)	0.173
LAD, mm, mean ± SD	45.19 ± 5.20	45.19 ± 5.44	0.999
LVEF, %, mean ± SD	54.81 ± 4.97	53.32 ± 5.73	0.026
D-dimer, μg/mL, median (IQR)	2.28 (1.20, 4.10)	2.37 (1.20, 5.30)	0.371
Creatinine, μmol/L, median (IQR)	71.20 (58.30, 83.00)	70.35 (56.0, 92.8)	0.882
Surgery-related baseline characteristics			
Symptom-to-Femoral Puncture Time, h, median (IQR)	3.00 (1.10, 5.00)	3.00 (1.50, 5.00)	0.831
Combined with IVT, *n* (%)	56 (49.56)	80 (43.72)	0.327
Surgical methods, *n* (%)			0.104
Pure mechanical thrombectomy	97 (85.84)	137 (74.86)	
Pure intra-arterial thrombolysis	7 (6.19)	23 (12.57)	
Combined dual procedures	7 (6.19)	21 (11.48)	
Combined multiple procedures	2 (1.77)	2 (1.09)	
mTICI ≥ 2b, *n* (%)	104 (92.04)	159 (86.89)	0.171
Combined with multiple vascular occlusions, *n* (%)	21 (18.58)	39 (21.31)	0.571
Combined with tandem vascular lesions, *n* (%)	6 (5.31)	9 (4.92)	0.881
Combined with intracranial atherosclerotic stenosis (>70%), *n* (%)	18 (15.93)	43 (23.50)	0.118
Cerebral infarct size, *n* (%)			0.002
>5 cm	29 (25.66)	58 (31.69)	
3–5 cm	57 (50.44)	109 (59.56)	
≤3 cm	27 (23.89)	16 (8.74)	
Postoperative HT, *n* (%)	14 (12.39)	58 (31.69)	<0.001

BMI, body mass index; TIA, transient ischemic attack; NIHSS, National institutes of health stroke scale; mRS, modified Rankin Scale; NT-proBNP, N-terminal pro-B-type natriuretic peptide; LAD, left atrial diameter; LVEF, left ventricular ejection fraction; IVT, intravenous thrombolysis; mTICI, modified thrombolysis in cerebral ischemia; HT, hemorrhagic transformation.

**Figure 1:**
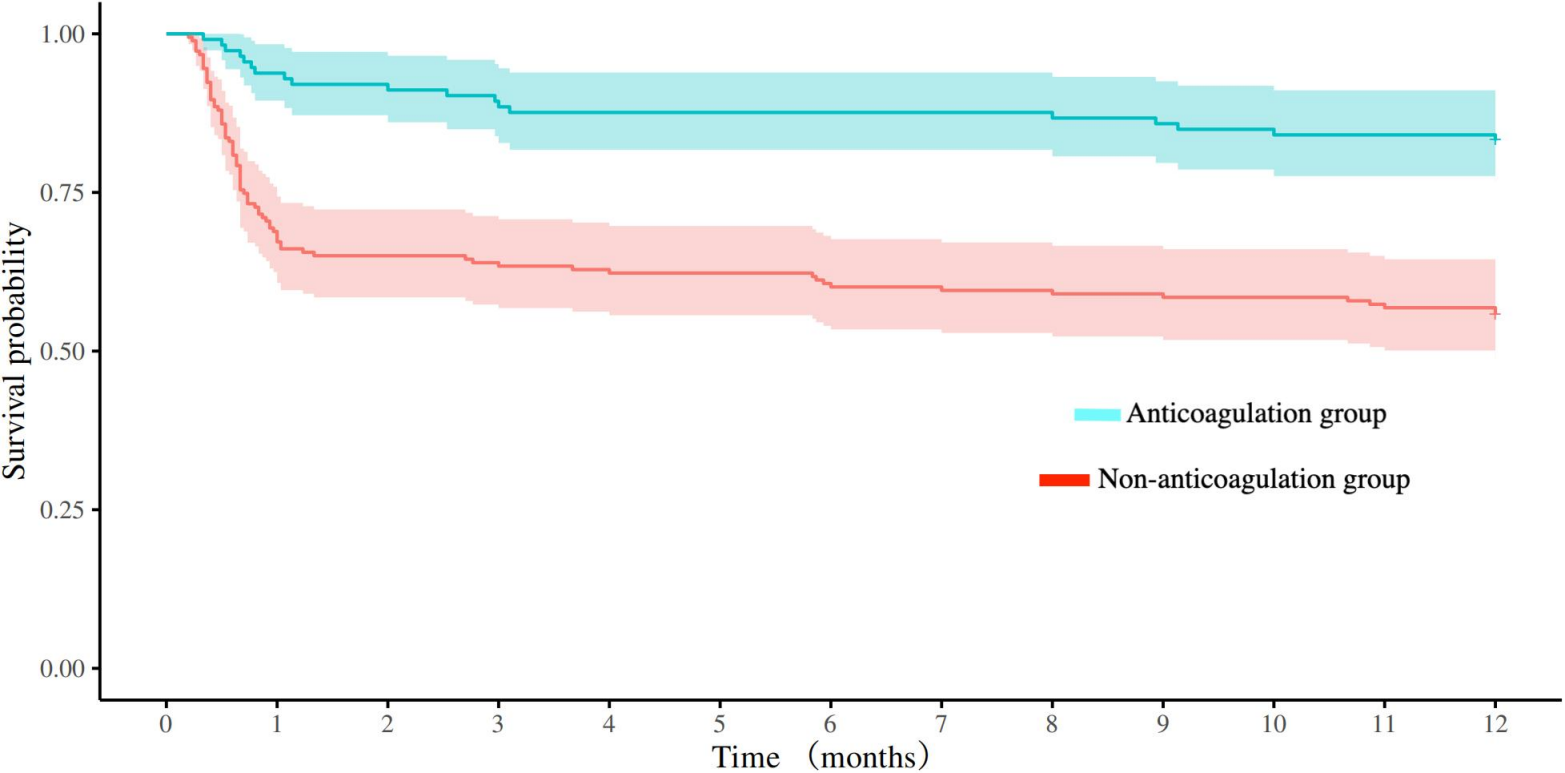
Comparison of survival curves between the anticoagulation and non-anticoagulation groups before propensity score matching.

Standardized follow-up was conducted at 90 days and 1 year after discharge. The anticoagulation group had significantly higher rates of favorable functional outcomes at both 90 days (mRS 0–2: 60.18% vs. 25.14%, *P* < 0.001) and 1 year (mRS 0–2: 64.60% vs. 30.05%, *P* < 0.001) compared to the non-anticoagulation group. The anticoagulation group had lower all-cause mortality compared with non-anticoagulated group at both 90-day (10.62% vs. 37.70%, *P* < 0.001) and 1-year follow-up (16.81% vs. 44.26%, *P* < 0.001). No significant intergroup disparities emerged in recurrent IS/SE or safety endpoints (Table 2). Kaplan–Meier survival analysis revealed superior cumulative survival in the anticoagulation group throughout the 1-year follow-up (log-rank *P* < 0.001), as shown in Figure 1.

**Table 2:** Prognostic outcomes between the anticoagulation and non-anticoagulation groups before PSM.

Prognostic outcomes, *n* (%)	Anticoagulation group (*n* = 113)	Non-anticoagulation group (*n* = 183)	*P* value
Favorable functional outcomes at 90 days (mRS 0–2)	68 (60.18)	46 (25.14)	<0.001
Favorable functional outcomes at 1 year (mRS 0–2)	73 (64.60)	55 (30.05)	<0.001
Recurrent IS/SE within 90 days	2 (1.77)	3 (1.64)	0.933
Safety outcomes within 90 days	2 (1.77)	0 (0.00)	0.071
All-cause mortality outcomes within 90 days	12 (10.62)	69 (37.70)	<0.001
Recurrent IS/SE within 1 year	5 (4.42)	15 (8.20)	0.209
Safety outcomes within 1 year	2 (1.77)	1 (0.55)	0.307
All-cause mortality outcomes within 1 year	19 (16.81)	81 (44.26)	<0.001

mRS, modified Rankin Scale; IS/SE, ischemic stroke/systemic embolism.

### 3.2 Propensity score matching analysis

#### 3.2.1 Baseline characteristics of patients after PSM

To reduce selection bias and the influence of confounding factors, propensity score matching (PSM) was applied. A total of 86 matched pairs were successfully obtained. After matching, the baseline characteristics were evenly distributed across the two groups, exhibiting no statistically significant differences, as shown in Table 3.

**Table 3:** Baseline characteristics of the anticoagulation vs. non-anticoagulation groups after PSM.

Characteristics	Anticoagulation group (*n* = 86)	Non-anticoagulation group (*n* = 86)	*P* value
Age, years, mean ± SD	72.92 ± 8.96	72.94 ± 9.20	0.987
Male, *n* (%)	52 (60.47)	50 (58.14)	0.756
BMI, kg/m^2^, mean ± SD	25.62 ± 3.23	24.72 ± 3.11	0.063
Medical history, *n* (%)			
Smoking	35 (40.70)	30 (34.88)	0.432
Drinking	23 (26.74)	22 (25.58)	0.862
Stroke/TIA	21 (24.42)	25 (29.07)	0.491
Hypertension	54 (62.79)	54 (62.79)	1
Diabetes mellitus	25 (29.07)	26 (30.23)	0.867
Coronary heart disease	15 (17.44)	9 (10.47)	0.187
Hyperlipemia	32 (37.21)	35 (40.70)	0.639
Heart failure	17 (19.77)	16 (18.60)	0.846
Types of AF, *n* (%)			
Paroxysmal AF (classification by duration)	14 (16.28)	15 (17.44)	0.839
Non-paroxysmal AF (classification by duration)	72 (83.72)	71 (82.56)	
KAF (classification by diagnosis time)	67 (77.91)	62 (72.09)	0.379
AFDAS (classification by diagnosis time)	19 (22.09)	24 (27.91)	
Clinically relevant baseline characteristics			
History of pre-stroke OAC use, *n* (%)	11 (12.79)	5 (5.81)	0.115
CHA2DS2-VASc score, median (IQR)	5.00 (4.00, 6.00)	5.00 (4.00, 6.00)	0.901
HAS-BLED score, median (IQR)	2.00 (2.00, 2.00)	2.00 (2.00, 2.00)	0.245
Admission NIHSS score, median (IQR)	16.00 (12.00, 20.00)	16.00 (12.00, 22.50)	0.698
Admission mRS score, median (IQR)	4.00 (4.00, 5.00)	4.00 (4.00, 5.00)	0.983
Discharge NIHSS score, median (IQR)	6.00 (2.80, 12.30)	7.50 (3.00, 13.30)	0.865
Discharge mRS score, median (IQR)	4.00 (2.00, 5.00)	3.00 (1.00, 5.00)	0.974
NT-proBNP, pg/mL, median (IQR)	1,453.64 (872.30, 2,347.30)	1,719.00 (758.90, 2,509.00)	0.781
LAD, mm, mean ± SD	45.41 ± 5.47	45.05 ± 5.29	0.673
LVEF, %, mean ± SD	53.90 ± 4.54	53.56 ± 5.44	0.659
D-dimer, μg/mL, median (IQR)	2.11 (1.20, 3.40)	2.06 (1.10, 3.70)	0.875
Creatinine, μmol/L, median (IQR)	71.60 (59.60, 86.30)	69.50 (55.80, 83.80)	0.367
Surgery-related baseline characteristics			
Symptom-to-Femoral Puncture Time, h, median (IQR)	3.00 (1.00, 4.50)	2.50 (1.50, 4.50)	0.742
Combined with IVT, *n* (%)	37 (43.02)	42 (48.84)	0.444
Surgical methods, *n* (%)			0.544
Pure mechanical thrombectomy	72 (83.72)	69 (80.23)	
Pure intra-arterial thrombolysis	7 (8.14)	6 (6.98)	
Combined dual procedures	5 (5.81)	10 (11.63)	
Combined multiple procedures	2 (2.33)	1 (1.16)	
mTICI ≥ 2b, *n* (%)	77 (89.53)	79 (91.86)	0.6
Combined with multiple vascular occlusions, *n* (%)	19 (22.09)	14 (16.28)	0.333
Combined with tandem vascular lesions, *n* (%)	4 (4.65)	7 (8.14)	0.35
Combined with intracranial atherosclerotic stenosis (>70%), *n* (%)	19 (22.09)	24 (27.91)	0.379
Cerebral infarct size, *n* (%)			0.088
>5 cm	22 (25.58)	33 (38.37)	
3–5 cm	44 (51.16)	42 (48.84)	
≤3 cm	20 (23.26)	11 (12.79)	
Postoperative HT, *n* (%)	13 (15.12)	9 (10.47)	0.361

BMI, body mass index; TIA, transient ischemic attack; NIHSS, National institutes of health stroke scale; mRS, modified Rankin Scale; NT-proBNP, N-terminal pro-B-type natriuretic peptide; LAD, left atrial diameter; LVEF, left ventricular ejection fraction; IVT, intravenous thrombolysis; mTICI, modified thrombolysis in cerebral ischemia; HT, hemorrhagic transformation.

#### 3.2.2 Prognostic outcomes of patients after PSM

**Figure 2:**
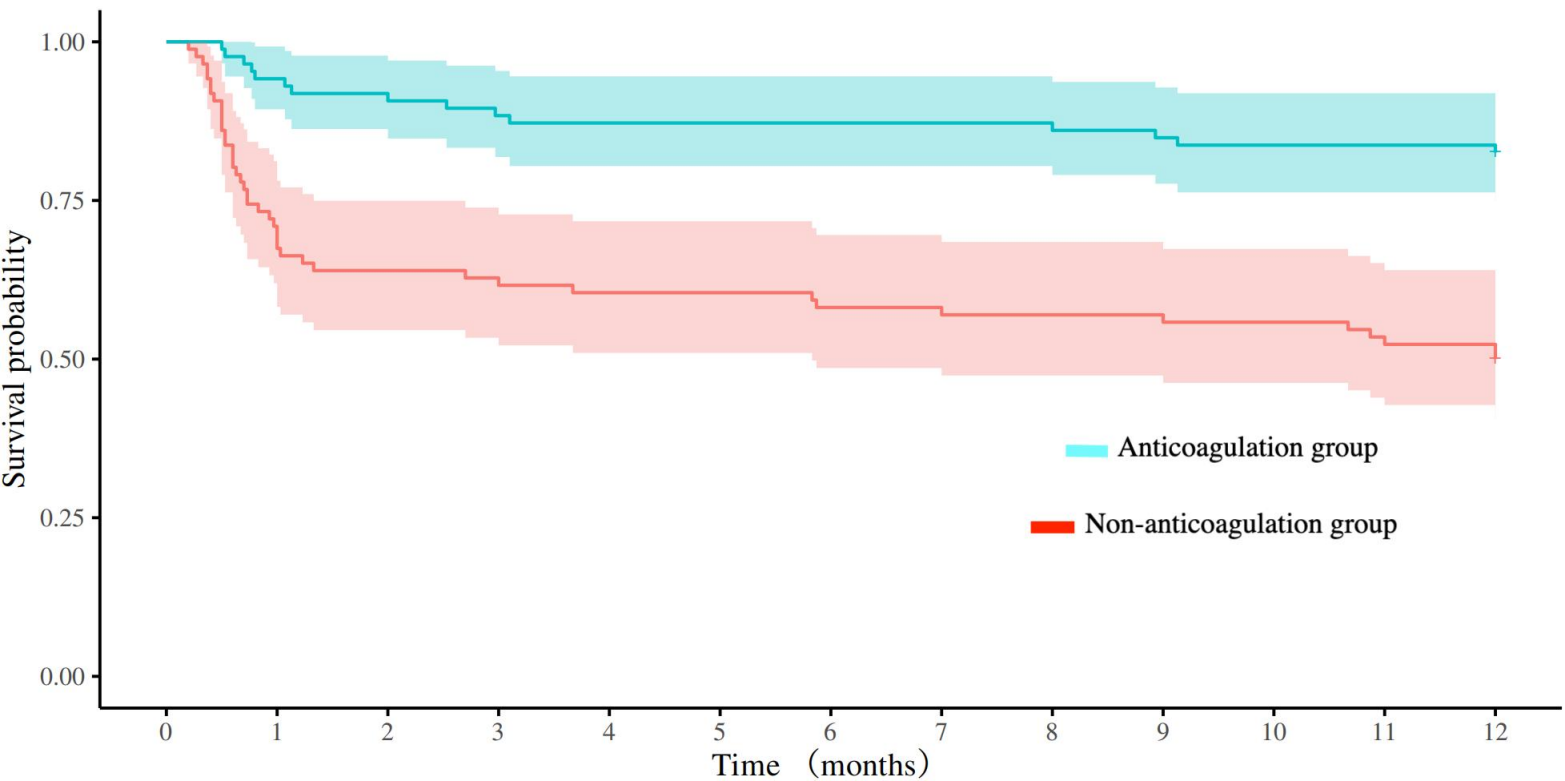
Comparison of survival curves between the anticoagulation and non-anticoagulation groups after propensity score matching.

**Figure 3:**
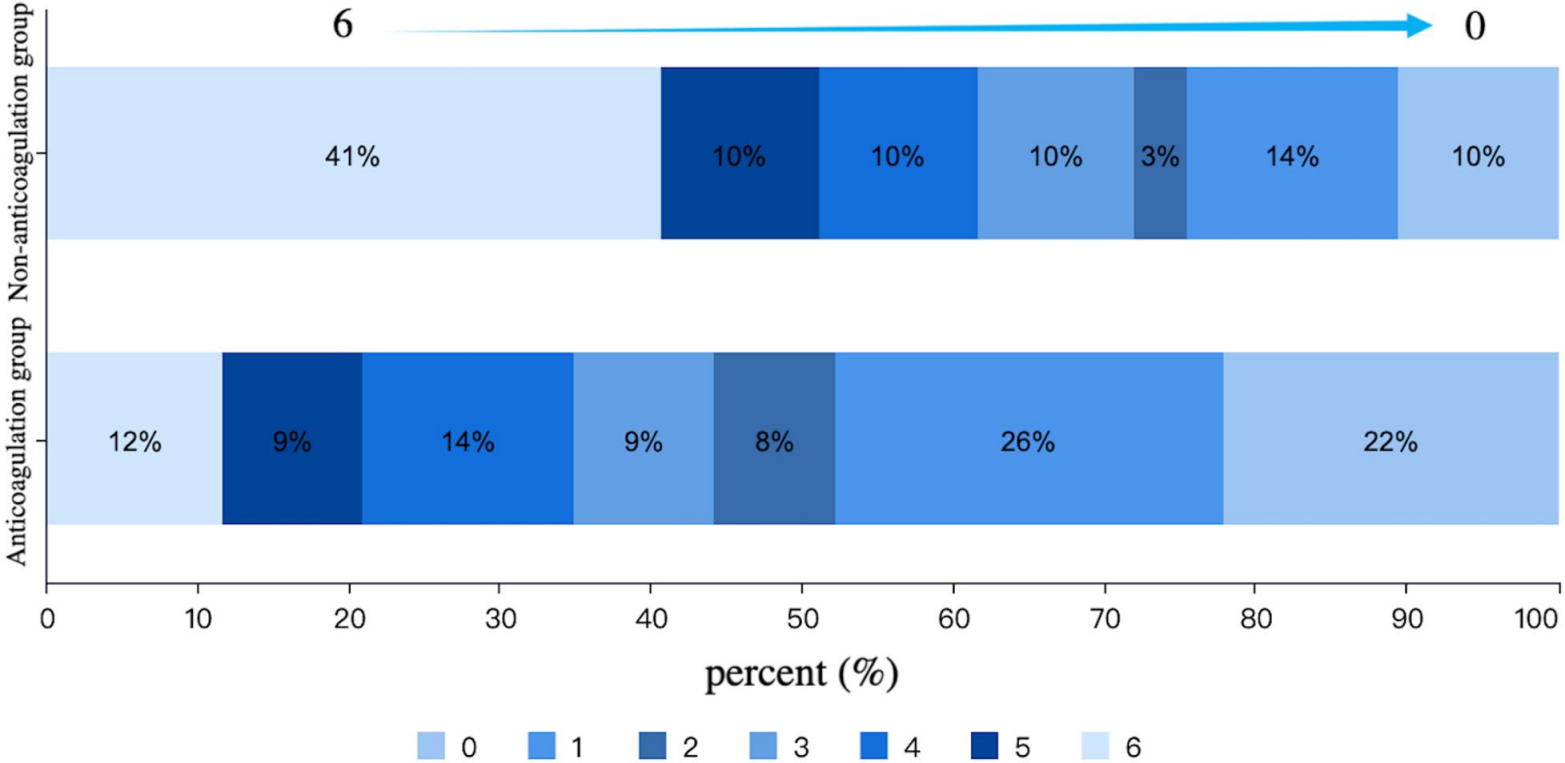
Distribution of mRS scores at 90-day follow-up in the anticoagulation and non-anticoagulation groups.

**Figure 4:**
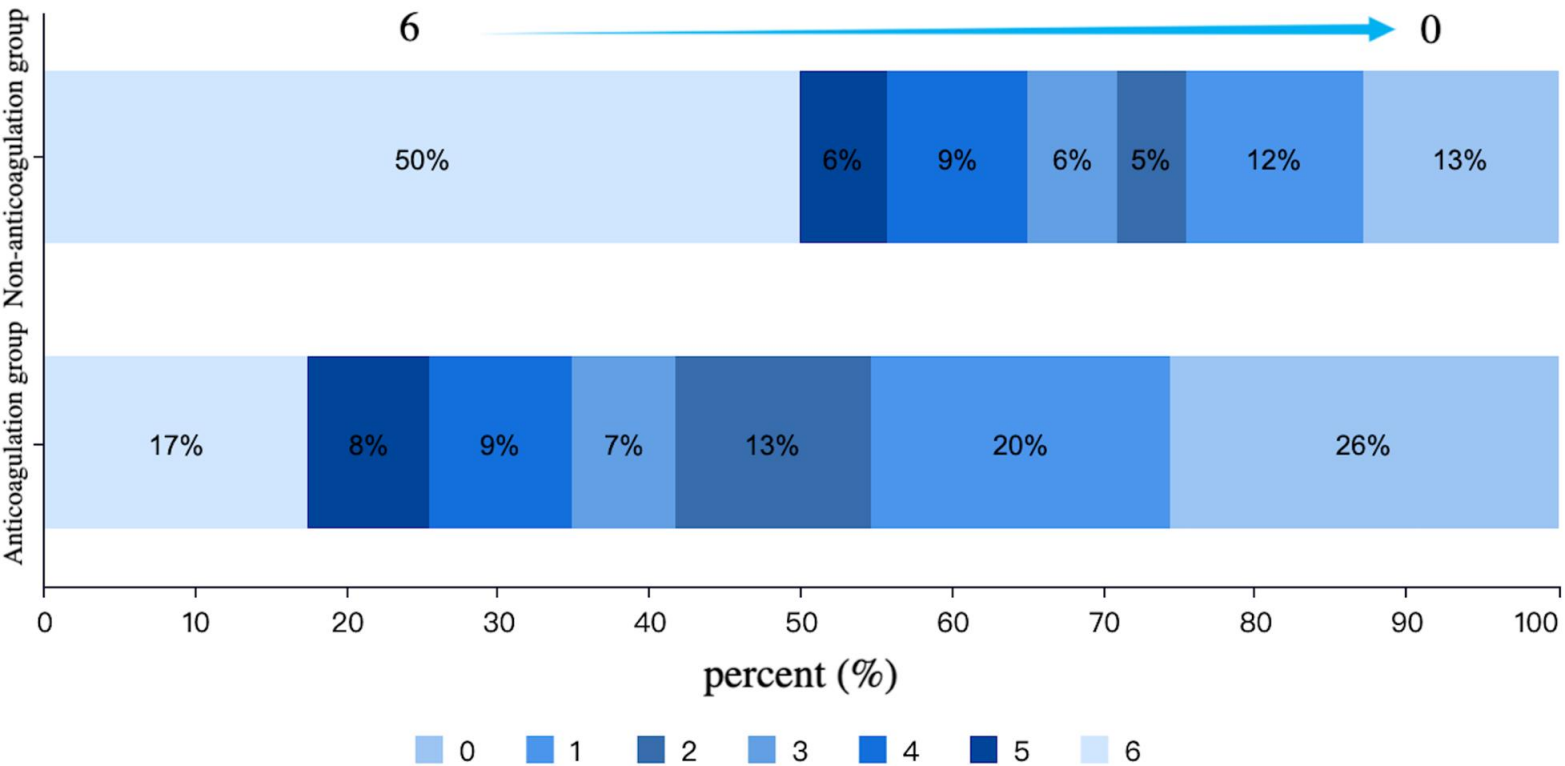
Distribution of mRS scores at 1-year follow-up in the anticoagulation and non-anticoagulation groups.

After PSM, there were 86 patients in both anticoagulation group and non-anticoagulation group. Comparative analysis revealed significantly higher rates of favorable functional outcomes in the anticoagulation group vs. non-anticoagulated group at both 90-day (55.81% vs. 27.91%, *P* < 0.001) and 1-year follow-up (60.47% vs. 30.23%, *P* < 0.001). The anticoagulation group had a lower all-cause mortality rate compared with non-anticoagulated group at both 90-day (11.63% vs. 40.70%, *P* < 0.001) and 1-year follow-up (17.44% vs. 50%, *P* < 0.001). No substantial disparities were seen between the two groups regarding recurrent IS/SE outcomes, safety outcomes, as shown in Table 4. The Kaplan–Meier curve based on the matched cohort demonstrated a higher survival rate in the anticoagulation group throughout the 1-year follow-up (log-rank *P* < 0.001), as shown in Figure 2. The distributions of mRS scores at the 90-day and 1-year follow-up marks are presented in Figures 3, 4, respectively.

**Table 4:** Prognostic outcomes between the anticoagulation and non-anticoagulation groups after PSM.

Prognostic outcomes, *n* (%)	Anticoagulation group (*n* = 86)	Non-anticoagulation group (*n* = 86)	*P* value
Favorable functional outcomes at 90 days (mRS 0–2)	48 (55.81)	24 (27.91)	<0.001
Favorable functional outcomes at 1 year (mRS 0–2)	52 (60.47)	26 (30.23)	<0.001
Recurrent IS/SE within 90 days	2 (2.33)	3 (3.49)	0.65
Safety outcomes within 90 days	2 (2.33)	0 (0.00)	0.155
All-cause mortality outcomes within 90 days	10 (11.63)	35 (40.70)	<0.001
Recurrent IS/SE within 1 year	4 (4.65)	6 (6.98)	0.515
Safety outcomes within 1 year	2 (2.33)	0 (0.00)	0.155
All-cause mortality outcomes within 1 year	15 (17.44)	43 (50.00)	<0.001

mRS, modified Rankin Scale; IS/SE, ischemic stroke/systemic embolism.

#### 3.2.3 Logistic regression analysis

After adjustment for potential confounders, the results of the multivariate analysis revealed that OAC treatment at discharge (anticoagulation group) was an independent factor affecting the prognostic function at 90 days (OR = 4.478, 95% CI: 1.122–17.874, *P* = 0.034) and the good prognostic function at 1 year (OR = 4.168, 95% CI: 1.118–5.542, *P* = 0.033), as shown in Table 5.

**Table 5:** Multivariate analysis of the impact of OAC therapy on prognostic functional outcomes.

Prognostic function outcome	Univariate analysis		Multivariate analysis	
	OR (95% CI)	*P* value	OR (95% CI)	*P* value
Favorable functional outcomes at 90 days (mRS 0–2)	3.263 (1.729–6.157)	<0.001	4.478 (1.122–17.874)	0.034
Favorable functional outcomes at 1 year (mRS 0–2)	3.529 (1.877–6.636)	<0.001	4.168 (1.118–5.542)	0.033

#### 3.2.4 Subgroup analysis

**Figure 5:**
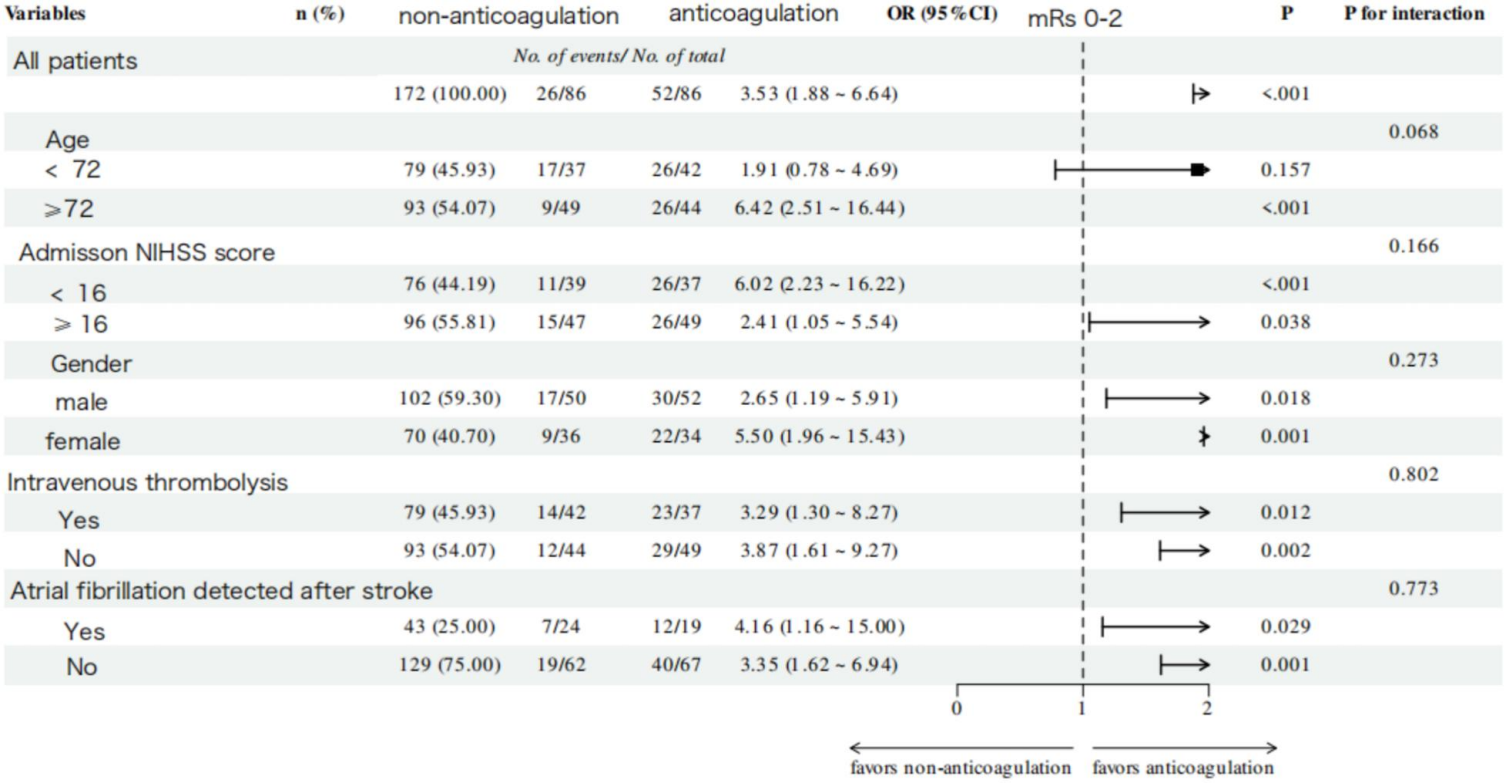
Subgroup analysis of favorable functional outcome at 1-year follow-up in the anticoagulation and non-anticoagulation groups.

**Figure 6:**
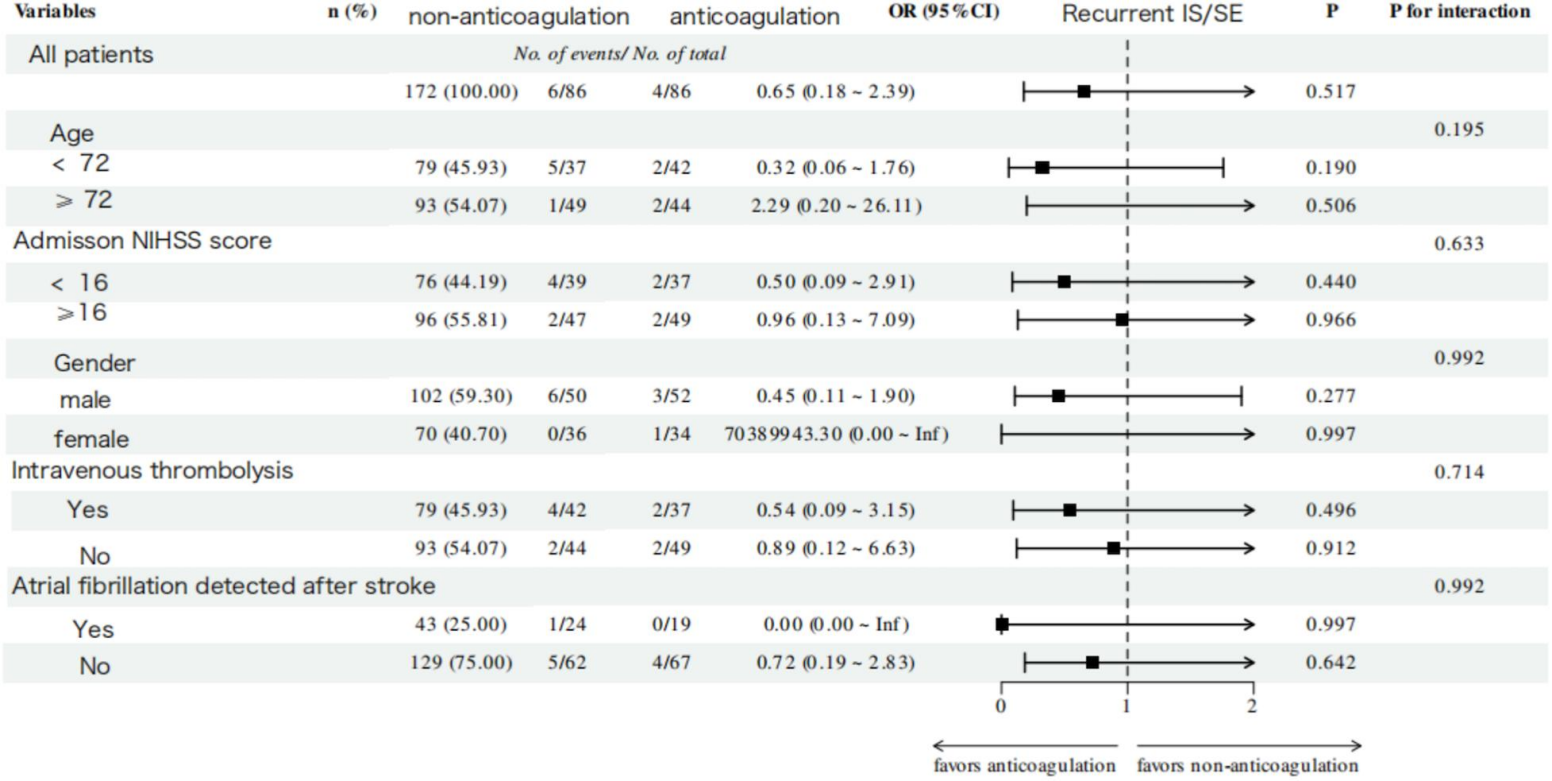
Subgroup analysis of recurrent ischemic stroke/systemic embolism outcome within 1 year of follow-up in the anticoagulation and non-anticoagulation groups.

No significant interaction effects were observed across all subgroups for both functional and efficacy outcomes. A consistently superior trend toward good functional outcomes was demonstrated in the anticoagulation group across all subgroups (*P* for trend < 0.001, Figure 5). Notably, this beneficial association was even more pronounced in specific subgroups, including patients aged ≥72 years (OR = 6.42, 95% CI: 2.51–16.44, *P* < 0.001), those with baseline NIHSS score < 16 (OR = 6.02, 95% CI: 2.23–16.22, *P* < 0.001), and females (OR = 5.50, 95% CI: 1.96–15.43, *P* < 0.001). For the efficacy outcome, OAC-treated patients demonstrated a trend toward a lower risk of IS/SE across all subgroups; however, this trend did not reach statistical significance (Figure 6).

## 4 Discussion

In AF-associated AIS, during hospitalization and post-discharge periods, timing of initiation of anticoagulation remains suboptimal (18), potentially attributable to heightened post-EVT hemorrhagic transformation risk and stroke severity. Prior research has demonstrated the incidence of HT in AIS is approximately 18–42 percent (19). During the acute phase of stroke, neuroinflammatory responses exacerbate blood-brain barrier injury, thereby increasing vascular permeability. Concurrently, rapid post-EVT cerebral reperfusion may facilitate the transmural passage of water and osmotic solutes across the compromised barrier, elevating hemorrhagic transformation risk (15). A meta-analysis of 25 cohort studies showed that age and a higher NIHSS score at admission were predictors of symptomatic intracranial hemorrhage (sICH) in patients with AIS after EVT (20). Tian et al. included 633 patients who received EVT treatment in their study and found that the multivariate model indicated that the baseline NIHSS score was an independent predictor of sICH after EVT (21). In this study, we also found that patients who did not receive OACs had higher HAS-BLED scores, higher NIHSS scores, higher proportion of postoperative HT in the hospital compared with those who received OAC at discharge, which may affect the use of OAC.

Existing research indicates that optimal anticoagulation strategies enhance long-term functional recovery while mitigating mortality and disability risks in stroke patients with AF (22, 23). Yan et al. conducted a study of 400 AF-associated AIS patients, demonstrating that in-hospital OAC initiation independently predicted superior functional recovery (mRS 0–1) and favorable neurological status (mRS 0–2) at 1-year assessment (24). Weller et al. analyzed 82 AF patients with acute large vessel occlusive stroke undergoing EVT and carotid stenting, demonstrating significantly elevated 90-day mortality rates in non-anticoagulated patients compared to discharged OAC recipients (25). In this study, there was a selection bias in the administration of anticoagulant therapy to stroke patients, as those who received it tended to have milder stroke severity, smaller infarct volumes, and a lower risk of hemorrhage. We employed PSM analysis to perform 1:1 matching between the two groups on some baseline characteristics potentially affecting prognostic outcomes, incorporating a total of nine matching factors. Among these, the admission NIHSS score, HAS-BLED score, LVEF, post-operative HT, infarct size, discharge NIHSS score, and the discharge mRS score were variables that showed statistically significant differences in baseline characteristics between the two groups (*P* < 0.05). Furthermore, the study was specifically restricted to patients with AF complicated by stroke who underwent EVT. It was necessary to control for two factors that could potentially influence the study results—AF subtype and surgical technique. Therefore, these were included as matching factors to minimize the disparity in confounding variables. Our results demonstrate that in a majority group of stroke patients without severe bleeding risk who were clinically selected for anticoagulation therapy, regular long-term anticoagulation was significantly associated with improved functional and mortality outcomes. This association remained significant after PSM adjusted for key between-group differences.

Patients in the anticoagulation group exhibited a significantly lower 1-year all-cause mortality in this study. Kaplan–Meier analysis revealed that the survival benefit associated with anticoagulant therapy primarily emerged within the first month after discharge. Acute-phase inflammatory and prothrombotic responses peak within 48–72 h post-stroke and can persist for several weeks (26). Initiating anticoagulation early in the post-EVT period might provide protection during this critical time window, thereby contributing to the observed reduction in all-cause mortality. In addition, the lack of observed difference in recurrent IS/SE events between the anticoagulation and non-anticoagulation groups in this study may be attributed to residual confounding by unmeasured factors, such as postoperative rehabilitation, frailty, and postoperative infection. In the non-anticoagulation group, a higher burden of these unmeasured negative prognostic factors likely contributed to more severe illness and higher short-term mortality. For some critically ill patients, the imminent risk of death may have precluded them from reaching the point where a stroke event could be observed, thereby obscuring a difference in effectiveness outcomes.

HT demonstrates higher incidence in AF patients with AIS undergoing EVT, predominantly manifesting as asymptomatic radiologic findings within 36 h post-procedure (15). Anticoagulation therapy needs to be restarted after individualized evaluation, and there is no specific time for initiation of anticoagulation. ELAN trial subanalyses demonstrated early direct oral anticoagulant initiation in HT patients did not significantly increase symptomatic intracranial hemorrhage incidence. However, functional outcomes may be compromised in those with parenchymal hematoma conversion (27). Escudero et al. conducted a multicenter analysis of 1,489 nonvalvular atrial fibrillation (NVAF) patients from the SITS registry cohort receiving acute stroke interventions [intravenous thrombolysis (IVT), EVT, or combined treatment]. Their findings demonstrated the safety profile of initiating dabigatran within 1–3 days post-stroke, with minimal thromboembolic or hemorrhagic events observed during 3-month follow-up across all treatment modalities (2.2%) (28). In this study, 72 patients developed HT post-EVT, but no clear classification study was carried out. Of these, 14 patients resumed OAC at discharge following multidisciplinary assessment. Comparative analysis demonstrated no significant between-group disparity in 1-year hemorrhagic risk despite comparable HT incidence. Notably, one non-anticoagulated patient receiving dual antiplatelet therapy (DAPT) experienced symptomatic intracranial hemorrhage during follow-up. Consequently, patients developing post-EVT HT necessitate meticulous evaluation of antithrombotic regimen-related bleeding risks, while the net clinical benefit of early anticoagulation initiation remains uncertain, the large-scale randomized trials remain essential to establish evidence-based OAC resumption timelines in this high-risk population.

AF can be divided into KAF and AFDAS depending on the time of diagnosis. In stroke involving the insula, cerebral ischemia and cell death may affect the autonomic nervous system and cause AF, so it is uncertain whether AFDAS is the result of brain injury or the cause of stroke associated with multiple neurogenic mechanisms, it remains unsubstantiated by relevant evidence (29). Patients with AFDAS demonstrate distinct vascular risk profiles, including significantly lower rates of both baseline comorbidities and subsequent stroke events than KAF (30). The patients with AFDAS may have a lower atrial burden, a smaller left atrium, and a higher LVEF than those with KAF, with no differences in age, sex, stroke severity, or mortality (31–33). Ryu et al. investigated the relationship between detection time and functional outcomes of AF after mechanical thrombectomy, and the results showed that AFDAS was positively correlated with functional independence (34). Another study demonstrated that KAF demonstrates an elevated HT risk vs. AFDAS, but without concomitant mortality risk elevation (35). OAC is recommended across all AF subtypes to mitigate recurrent stroke risk. However, the net clinical benefit of OAC therapy in patients with AFDAS remains uncertain, because patients with AFDAS tend to exhibit more benign features. In this study, whether AF was AFDAS showed no significant interaction with functional or efficacy outcomes. Instead, anticoagulation was consistently linked to better functional outcomes across all AF subtypes. This suggests that early anticoagulation may confer a prognostic benefit regardless of the specific atrial fibrillation subtype. Therefore, more data from randomized controlled trials are warranted to determine the risk-benefit ratio of anticoagulation therapy in AFDAS populations.

## 5 Limitations

This study had several limitations. First, it was a single-center design with a limited sample size, which may introduce selection bias. Second, this study did not perform a detailed analysis of OAC regimens, including specific drug types and dosage adjustments. Third, while we assessed the impact of timely OAC initiation at discharge, we did not evaluate longitudinal changes in anticoagulation therapy during the 1-year follow-up period. Notably, patients with irregular medication adherence or delayed post-discharge OAC initiation were excluded from analysis. Fourth, we acknowledged that PSM may not have eliminated all selection bias and confounding, including differences in frailty, fall risk, baseline cognitive function, and independence. Future randomized controlled trials are needed to confirm these observations. Consequently, our findings primarily reflect the prognostic benefits of early, guideline-adherent anticoagulation in compliant patients. Thus, further randomized controlled trials are warranted to validate these observations.

## 6 Conclusion

This study offers novel real-world evidence supporting the role of post-EVT anticoagulation therapy in AF-associated AIS. Analysis indicates that among stroke patients clinically selected for anticoagulation therapy and without high risk of severe bleeding, OAC therapy is associated with improved functional and mortality outcomes compared with those not receiving OAC. The benefit remained statistically significant following PSM to adjust for intergroup disparities.

## Data Availability

The original contributions presented in the study are included in the article/Supplementary Material, further inquiries can be directed to the corresponding author.
