# Supplementary material for: Impact of anticoagulation management following endovascular therapy on prognosis of patients with atrial fibrillation and acute ischemic stroke
Source: Front Cardiovasc Med. 2026 Jan 15;12:1622665. doi: 10.3389/fcvm.2025.1622665 (PMC12852355; doi:10.3389/fcvm.2025.1622665)
Supplement: Supplementary file 1 [file Datasheet1.pdf]

Supplementary data：

Table S1 Initiation Timing of Anticoagulation Therapy in Stroke Patients from the Anticoagulation Group.

	Stroke severity classification	Initiation timing of anticoagulation therapy after stroke, days, median (IQR)
Before PSM	Mild (admission NIHSS score <8, n=17)	5.75 (3.57,8.37)
	Moderate (admission NIHSS score 8-15, n=35)	7.00 (4.54,12.00)
	Severe (admission NIHSS score >15, n=61)	8.50 (5.38,12.54)
After PSM	Mild (admission NIHSS score <8, n=13)	6.00 (4.19,8.85)
	Moderate (admission NIHSS score 8-15, n=27)	7.17 (4.54,14.42)
	Severe (admission NIHSS score>15, n=46)	9.20 (6.42,13.72)

Abbreviation: PSM, propensity score matching； NIHSS, National institutes of health stroke scale;

Table S2 Types and proportions of anticoagulant medications in the anticoagulation group.

	OAC type	Monotherapy	OAC combined with antiplatelet therapy
Before PSM	Edoxaban	6 (5.31)	0
	Rivaroxaban	52 (46.02)	16 (14.16)
	Dabigatran Etexilate	35 (30.97)	4 (3.54)
After PSM	Edoxaban	4 (4.65)	0
	Rivaroxaban	41 (47.67)	11 (12.79)
	Dabigatran Etexilate	27 (31.40)	3 (3.49)

Abbreviation: PSM, propensity score matching；OAC, oral anticoagulation;

Table S3 Proportion of patients receiving antiplatelet therapy in the non-anticoagulation group.

	SAPT	DAPT	No antiplatelet medication
Before PSM	68 (37.16)	48 (26.23)	67 (36.61)
After PSM	33 (38.37)	13 (15.12)	40 (46.51)

Abbreviation: SAPT, Single antiplatelet therapy; DAPT, Dual antiplatelet therapy; PSM，propensity score matching；
